# Supplementary material for: Trypanosoma brucei cattle infections contain cryptic transmission-adapted bloodstream forms at low parasitaemia
Source: Nat Commun. 2025 Nov 5;16:9776. doi: 10.1038/s41467-025-64750-y (PMC12589498; doi:10.1038/s41467-025-64750-y)
Supplement: Supplementary file 1 — Supplementary Information [file 41467_2025_64750_MOESM1_ESM.pdf]

**Supplementary Information File**

*Trypanosoma brucei* cattle infections contain cryptic transmission-adapted bloodstream forms at low parasitaemia

Stephen D. Larcombe[1], Edith Paxton[2], Christina Vrettou[2], Pieter C. Steketee[2], Keith R. Matthews[1], Liam J. Morrison[2], Emma M. Briggs[1,3]*

**Affiliations**
1. Institute for Immunology and Infection Research, School of Biological Sciences, Ashworth Laboratories, University of Edinburgh, Edinburgh, EH9 3FL, UK
2. Roslin Institute, Royal (Dick) School of Veterinary Studies, University of Edinburgh, Easter Bush, Midlothian, EH25 9RG, UK
3. Biosciences Institute, Faculty of Medical Sciences, Cookson Building, Newcastle University, Framlington Place, Newcastle upon Tyne, NE2 4HH, UK

*Correspondence to emma.briggs@newcastle.ac.uk

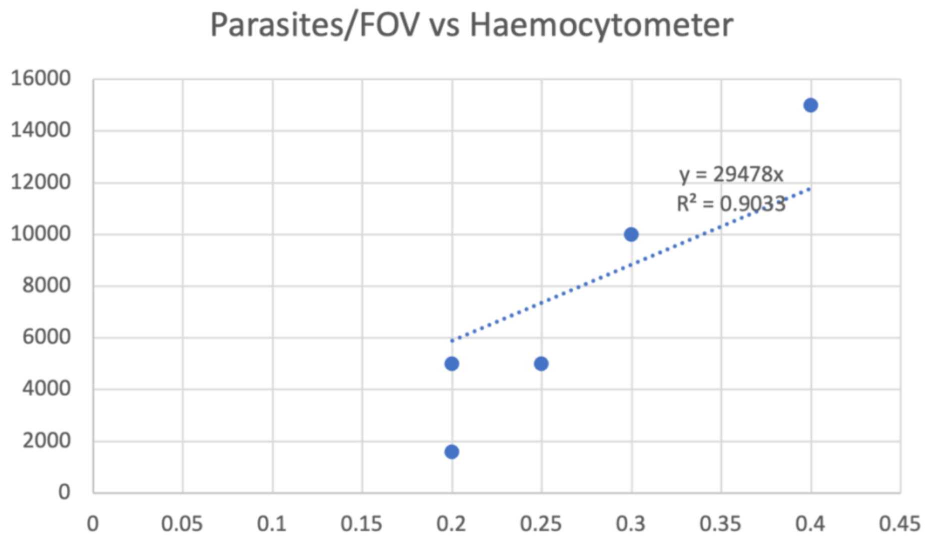


**Supplementary figure 1**. Estimating parasitaemia in cattle is difficult at low parasitaemia. In order to create a consistent score for parasitaemia, including periods when blood parasitaemia was below the limit of detection in whole blood and only apparent by examining parasites enriched by use of buffy coat preparations, we used a conversion derived from the linear relationship between parasites counted per ml of whole blood or per field of view (FOV) of purified buffy coat, for a subset of counts made at very low parasitaemia. The graph shows the strong linear relationship where the $R^2$ of the line is 0.9, and the resultant conversion is provided by the formula Parasites/ml = 29478 x Parasites/buffy coat FOV. Source data are provided as a Source Data file.

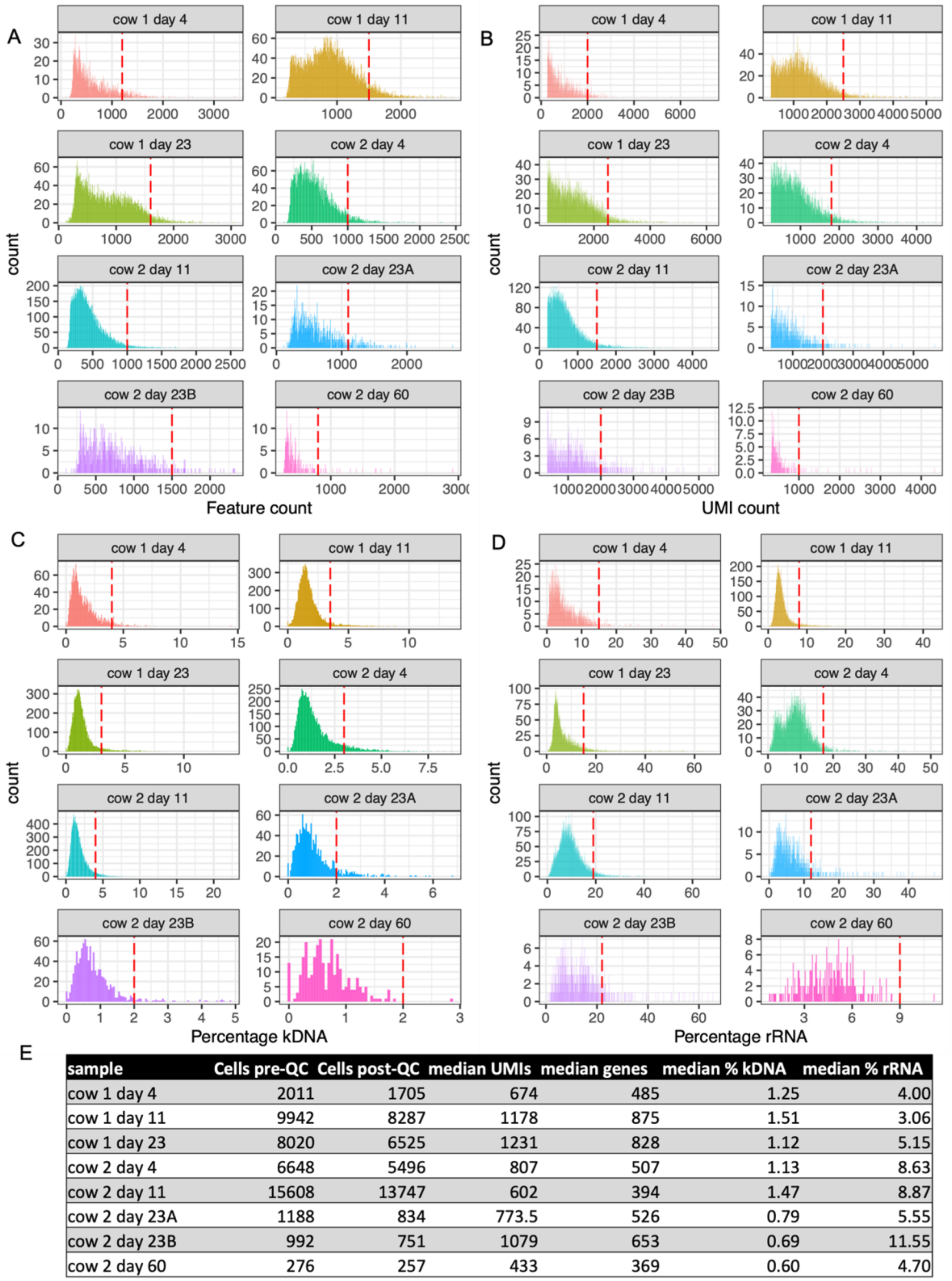


E

| sample | Cells pre-QC | Cells post-QC | median UMIs | median genes | median % kDNA | median % rRNA |
|---|---|---|---|---|---|---|
| cow 1 day 4 | 2011 | 1705 | 674 | 485 | 1.25 | 4.00 |
| cow 1 day 11 | 9942 | 8287 | 1178 | 875 | 1.51 | 3.06 |
| cow 1 day 23 | 8020 | 6525 | 1231 | 828 | 1.12 | 5.15 |
| cow 2 day 4 | 6648 | 5496 | 807 | 507 | 1.13 | 8.63 |
| cow 2 day 11 | 15608 | 13747 | 602 | 394 | 1.47 | 8.87 |
| cow 2 day 23A | 1188 | 834 | 773.5 | 526 | 0.79 | 5.55 |
| cow 2 day 23B | 992 | 751 | 1079 | 653 | 0.69 | 11.55 |
| cow 2 day 60 | 276 | 257 | 433 | 369 | 0.60 | 4.70 |

**Supplementary figure 2.** Metadata associated with scRNA-seq samples. Histograms showing the number of genes for which transcripts are detected (features, A), unique transcripts (UMIs, B), percentage of transcripts expressed from the mitochondrial kinetoplast maxicircle genome (Percentage kDNA, C) and percentage of transcripts encoding ribosomal RNA (Percentage rRNA, D), per parasite transcriptome. Red dashed lines indicate quality control thresholds used. E) Summary table of QC measures.

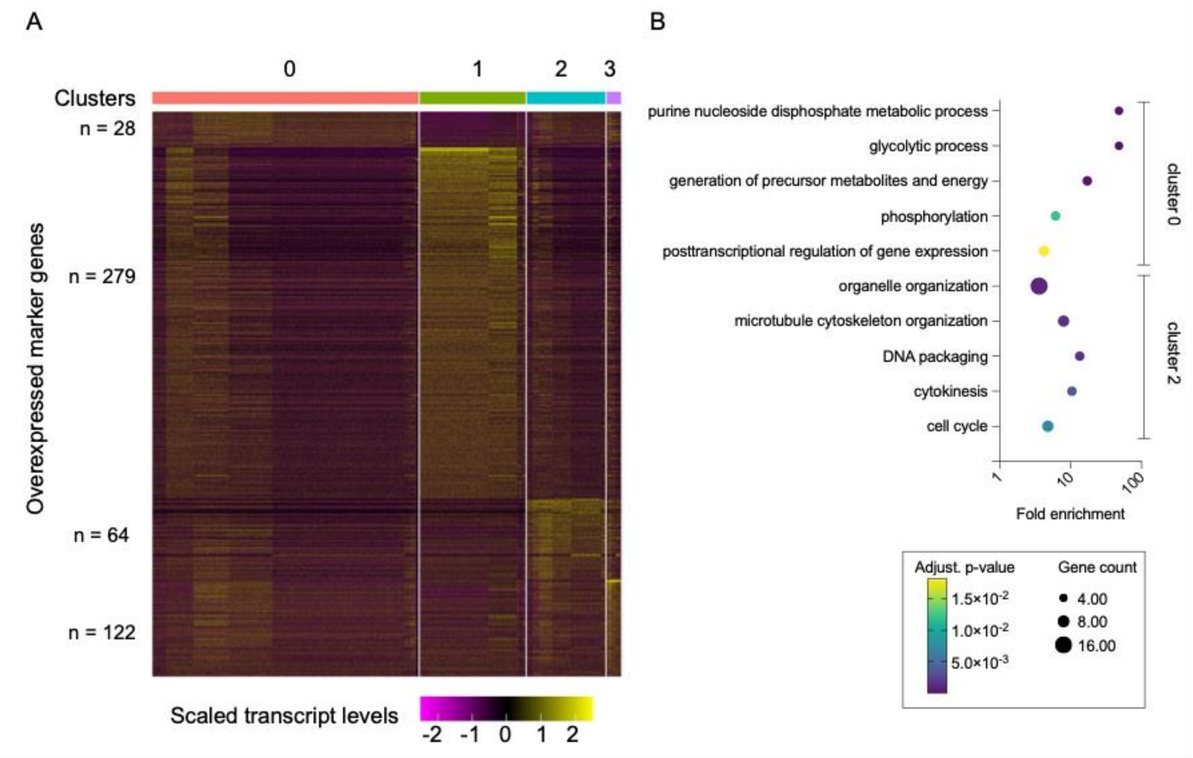


**Supplementary figure 3.** Gene markers of parasite clusters and associated GO terms. A) Heatmap shows scaled transcript levels for each marker genes separated per cluster. The number of markers for each cluster is indicated on the y-axis and cluster on the x-axis. B) Enriched biological process GO terms for cluster 0 and cluster 2. Plot indicated fold enrichment (x-axis), number of genes for each term (size) and the adjusted p-value (colour scheme) for each term.  Adjusted p-values are Benjamini-Hochberg adjusted p-values from Fisher's exact test.

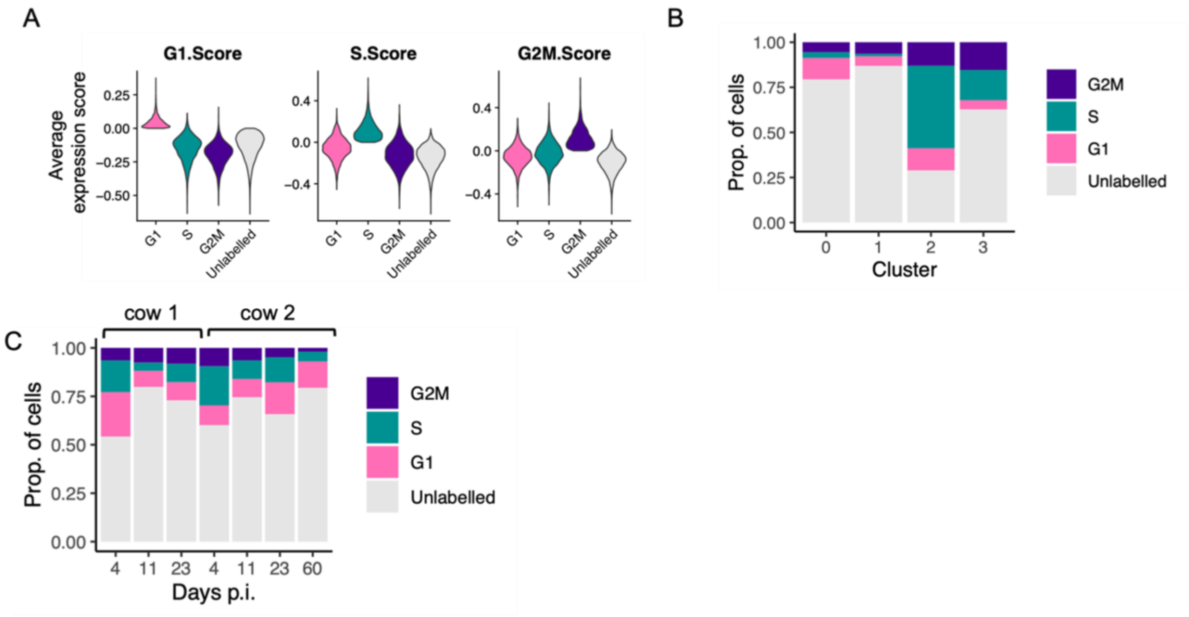


**Supplementary figure 4.** Cell cycle status of *T. brucei* in the cattle bloodstream inferred by transcriptome**.** A) Average gene expression score for Early G1, Late G1, S and G2/M phase marker genes. Scores below 0 indicate no over expression of marker gene set compared to control gene set. B) Proportion of cells in each phase or Unlabelled per cluster. C) Proportion of each phase or unlabelled per sample. Source data are provided as a Source Data file.

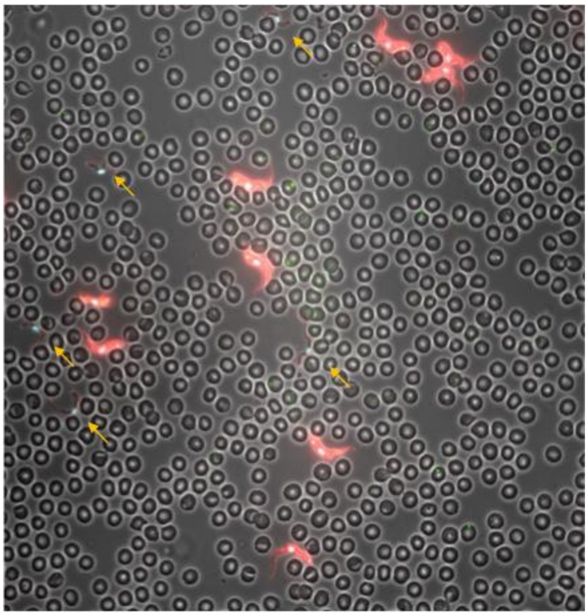

**Supplementary figure 5. Representative stumpy and slender forms from a mixed bloodstream form population from 7 days post-infection of a mouse.** Merged immunofluorescence microscopy image of a blood smear sample from a mouse infected with AnTat 1.1 90:13 pleomorphic *T. brucei* 7 days post infections, showing both stumpy and slender cells stained with the same αPAD1 antibody (red). Stumpy forms have positive surface PAD1 signal, whereas non-stumpy forms (slender or intermediate) show only weaker flagella staining (yellow arrows). DAPI staining is shown in cyan.

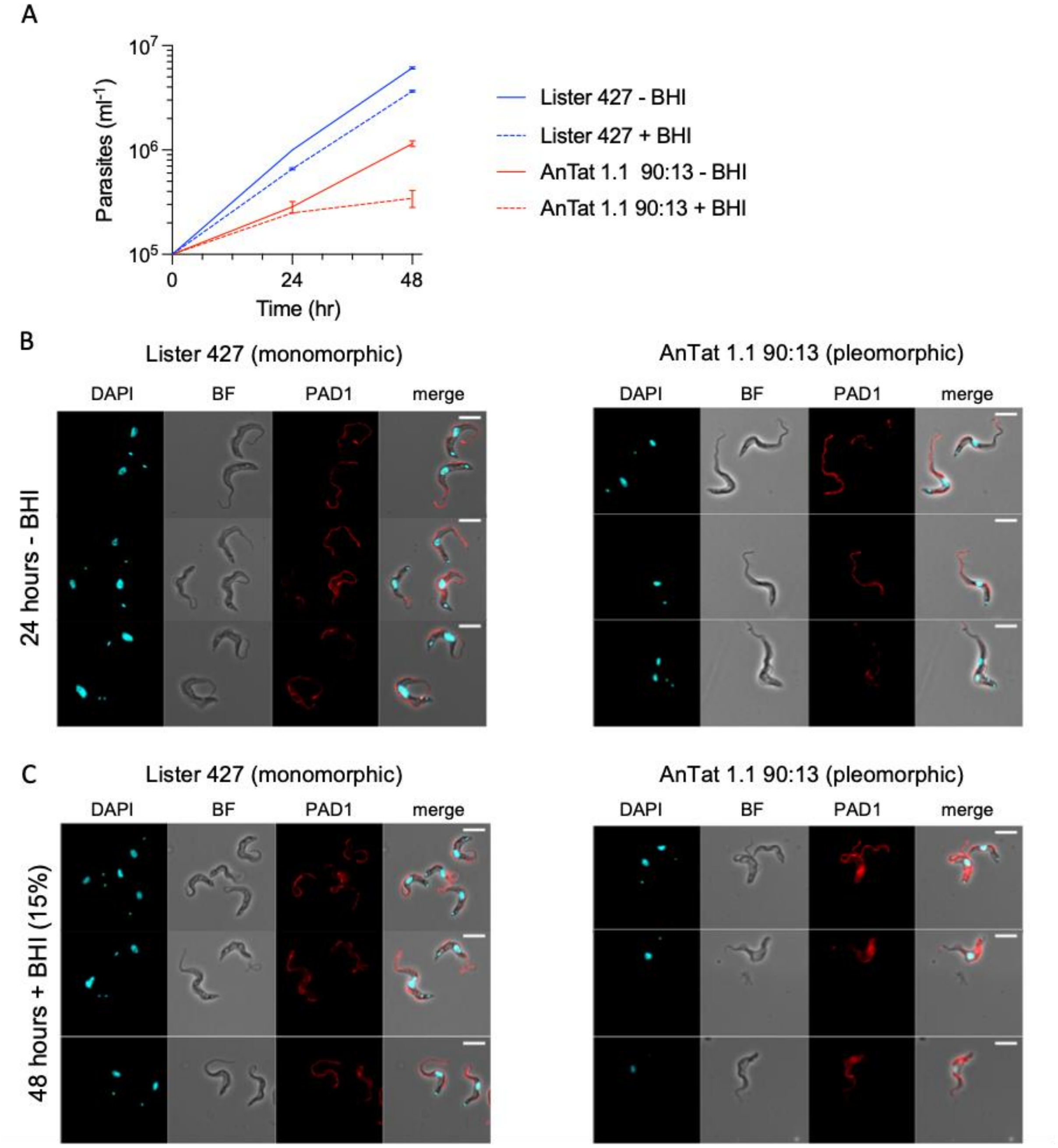


**Supplementary figure 6. PAD1 antibody staining on the flagella only does not indicate differentiation**. A) Growth Curves of Lister 427 (monomorphic) or AnTat 1.1 90:13 (pleomorphic) *T. brucei* strains grown continuously in HMI-9 in the presence or absence of BHI at 15%. B) Representative images showing staining with αPAD1 antibody of Lister 427 cells and AnTat cells at 24 hours in the absence of BHI. All cells have the same signal restricted to the flagella C) Representative images showing staining with αPAD1 antibody of Lister 427 cells and AnTat cells at 48 hours in the presence of BHI, showing only a subset of AnTat cells have developed a signal on the cell surface away from the flagella, these are absent in monomorphs suggesting only non-flagella localisation of the αPAD1 antibody is indicative of differentiation and flagella signal only is an artefact. Source data are provided as a Source Data file.

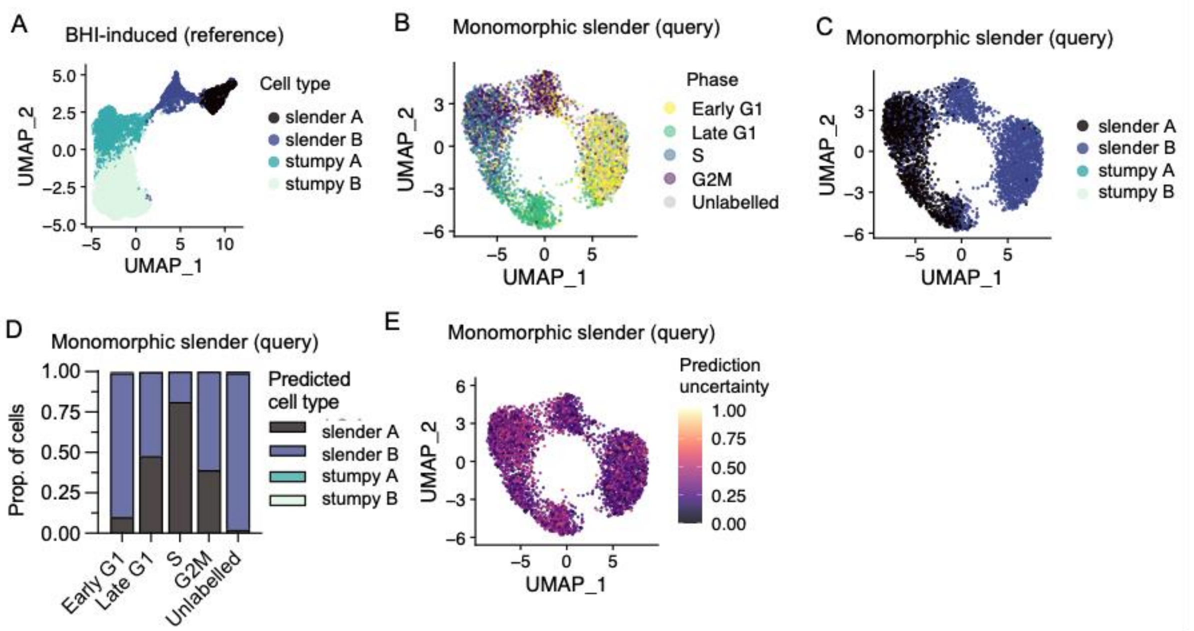


**Supplementary figure 7**. Cell type prediction correctly identifies slender parasites across datasets**.** A) UMAP of previously described scRNA-seq analysis of *T. brucei* treated with 10% brain heart infusion broth *in vitro* to generate slender and stumpy forms [1]. B) UMAP of monomorphic slender forms *T. brucei* replicating in culture [2] used as query data, coloured by cell cycle phase. Monomorphs are unable to form arrested stumpy forms. C) UMAP of monomorphic slender forms coloured by predicted cell type. Labels and colours are consistent with reference data in a. D) Proportions of each monomorphic slender cell cycle phase (x-axis) as shown in 2b, predicted to be *in vitro* generated cell-types. E) UMAP of monomorphic slender form transcriptomes coloured by uncertainty in cell type predictions. 0 indicates low prediction uncertainty and 1 indicates high uncertainty. Source data are provided as a Source Data file.

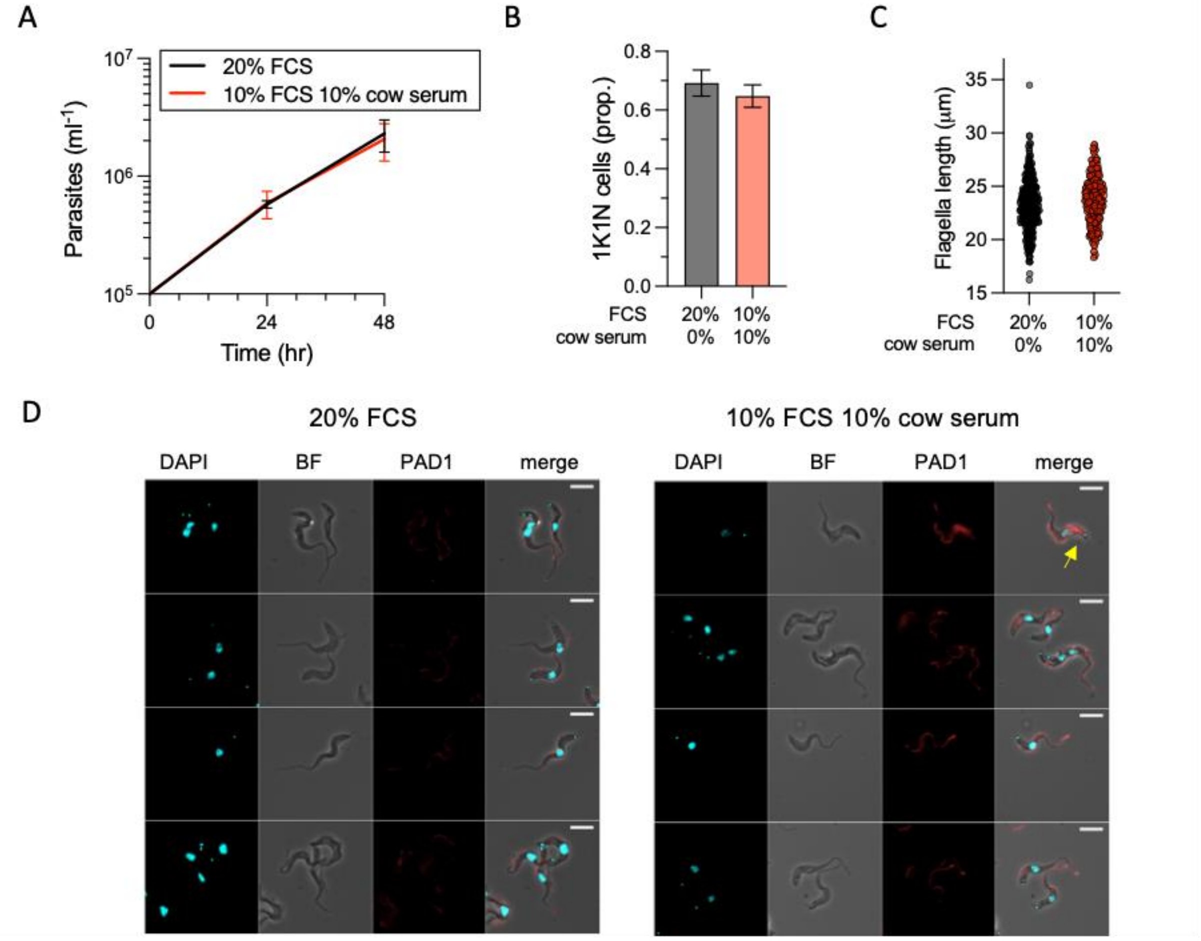


**Supplementary figure 8. Growth of cultured AnTat 1.1 90:*13 T. brucei* in cow serum does not generate parasites with the characteristics of bloodstream forms isolated from the cattle bloodstream**. A) Growth of AnTat 1.1 90:13 *T. brucei* cells grown continuously for 48 hr, following a week-long culture period for habituation to either 20% FCS (normal culture conditions), or a mix of 10% FCS and 10% cow serum. No cultures could be adapted to only 20% cow serum. B) Flagella lengths and C) KN configurations of the cells from three replicates of each serum condition tested after 48 hours (high density). D) Representative images showing staining with αPAD1 antibody of *T. brucei* in either serum culture condition. FCS only cells had no surface PAD1 signal, 7 of 245 (2.8%) of cells grown in the presence of cow serum (10%) were PAD1 positive and these cells showed some stumpy morphology (yellow arrow). Source data are provided as a Source Data file.

1 Briggs, E. M., Rojas, F., McCulloch, R., Matthews, K. R. & Otto, T. D. Single-cell transcriptomic analysis of bloodstream Trypanosoma brucei reconstructs cell cycle progression and developmental quorum sensing. *Nat Commun* **12**, 5268 (2021).

2 Briggs, E. M. *et al.* Profiling the bloodstream form and procyclic form Trypanosoma brucei cell cycle using single-cell transcriptomics. *Elife* **12** (2023).
