# Supplementary material for: Trypanosoma brucei cattle infections contain cryptic transmission-adapted bloodstream forms at low parasitaemia
Source: Nat Commun. 2025 Nov 5;16:9776. doi: 10.1038/s41467-025-64750-y (PMC12589498; doi:10.1038/s41467-025-64750-y)
Supplement: Supplementary file 2 — Description Of Additional Supplementary File [file 41467_2025_64750_MOESM2_ESM.pdf]

Description of Additional supplementary files

Supplementary data 1.

Cluster markers (tab 1) and associated GO terms (tabs 2-5) for cattle data clusters 0-3 presented in Figure 1B.

Supplementary data 2.

Results of differential expression analysis after pseudo-bulking of scRNA-seq data from mouse, cow and in vitro experiments presented in Figure 4. Tab 1: Differential expression between mouse derived and BHI treated in vitro T. brucei samples. Tab 2: Differential expression between cow derived and BHI treated in vitro T. brucei samples. Tab 3: Differential expression between cow derived and mouse derived T. brucei samples. Tab4: Differential expression of ESAG genes between cow and mouse derived samples. Tab 5: Differential expression of ESAG4/GRESG4 genes. Tab 6: GO term enrichment analysis of genes upregulated in cattle samples vs mouse samples. Tab 7: GO term enrichment analysis of genes upregulated in mouse samples vs cattle samples.

Supplementary data 3.

Fold change levels of slender and stumpy/stumpy-like marker gene transcripts in each dataset (cow, mouse and in vitro) presented in Figure 7.

Supplementary video 1.

Selection of live videos of T. brucei parasites isolated from either cow 1 or cow 2 in the chronic infection stage (days 51 onwards).
