# Supplementary material for: Trypanosoma brucei cattle infections contain cryptic transmission-adapted bloodstream forms at low parasitaemia
Source: Nat Commun. 2025 Nov 5;16:9776. doi: 10.1038/s41467-025-64750-y (PMC12589498; doi:10.1038/s41467-025-64750-y)
Supplement: Supplementary file 7 — Reporting summary [file 41467_2025_64750_MOESM7_ESM.pdf]

nature portfolio

Corresponding author(s): Dr Emma Briggs

Last updated by author(s): Sep 19, 2025

Reporting Summary

Nature Portfolio wishes to improve the reproducibility of the work that we publish. This form provides structure for consistency and transparency in reporting. For further information on Nature Portfolio policies, see our Editorial Policies and the Editorial Policy Checklist.

Statistics

For all statistical analyses, confirm that the following items are present in the figure legend, table legend, main text, or Methods section.

n/a	Confirmed	
☐	☒	The exact sample size (*n*) for each experimental group/condition, given as a discrete number and unit of measurement
☐	☒	A statement on whether measurements were taken from distinct samples or whether the same sample was measured repeatedly
☐	☒	The statistical test(s) used AND whether they are one- or two-sided<br>*Only common tests should be described solely by name; describe more complex techniques in the Methods section.*
☒	☐	A description of all covariates tested
☐	☒	A description of any assumptions or corrections, such as tests of normality and adjustment for multiple comparisons
☐	☒	A full description of the statistical parameters including central tendency (e.g. means) or other basic estimates (e.g. regression coefficient) AND variation (e.g. standard deviation) or associated estimates of uncertainty (e.g. confidence intervals)
☐	☒	For null hypothesis testing, the test statistic (e.g. *F*, *t*, *r*) with confidence intervals, effect sizes, degrees of freedom and *P* value noted<br>*Give P values as exact values whenever suitable.*
☒	☐	For Bayesian analysis, information on the choice of priors and Markov chain Monte Carlo settings
☒	☐	For hierarchical and complex designs, identification of the appropriate level for tests and full reporting of outcomes
☐	☒	Estimates of effect sizes (e.g. Cohen's *d*, Pearson's *r*), indicating how they were calculated

Our web collection on statistics for biologists contains articles on many of the points above.

Software and code

Policy information about availability of computer code

Data collection	N/A
Data analysis	Sequence mapping was performed with Cellranger v7.0.0. Single cell transcriptomics analysis was performed using Seurat software v 4.3.0.1, R v 4.4.0, scran v1.28.2, scater v1.29.4, singlecellexperiment v1.22.0 and DESeq2. Approaches have been previously published and are referenced. Code is available at Zenodo DOI:10.5281/zenodo.14515536. All tools are published and open source.

For manuscripts utilizing custom algorithms or software that are central to the research but not yet described in published literature, software must be made available to editors and reviewers. We strongly encourage code deposition in a community repository (e.g. GitHub). See the Nature Portfolio guidelines for submitting code & software for further information.

Data

Policy information about availability of data

All manuscripts must include a data availability statement. This statement should provide the following information, where applicable:

- Accession codes, unique identifiers, or web links for publicly available datasets
- A description of any restrictions on data availability
- For clinical datasets or third party data, please ensure that the statement adheres to our policy

All scRNA-seq sequences is deposited on the European Nucleotide Archive under study accession number PRJEB66078

Research involving human participants, their data, or biological material

Policy information about studies with human participants or human data. See also policy information about sex, gender (identity/presentation), and sexual orientation and race, ethnicity and racism.

Reporting on sex and gender	N/A
Reporting on race, ethnicity, or other socially relevant groupings	N/A
Population characteristics	N/A
Recruitment	N/A
Ethics oversight	N/A

Note that full information on the approval of the study protocol must also be provided in the manuscript.

Field-specific reporting

Please select the one below that is the best fit for your research. If you are not sure, read the appropriate sections before making your selection.

☒ Life sciences ☐ Behavioural & social sciences ☐ Ecological, evolutionary & environmental sciences

For a reference copy of the document with all sections, see nature.com/documents/nr-reporting-summary-flat.pdf

Life sciences study design

All studies must disclose on these points even when the disclosure is negative.

Sample size	Two cattle were selected at the sufficient number for infection as T. brucei Antat 1.1 infection is known to be efficient (100% of 14 cattle in pervious studies). Previous studies highlighted how many time points in the infection will have high enough parasite numbers for single cell RNA-seq which dictated the selection of 3 time points plus a final time point at the end of experiment when a larger sample could be collected. Selection of samples for microscopy in late infection was at the maximum permitted under the HSE animal licence. The number of parasites analysed was the maximum that could be obtained. Variable conditions other than a time course across infection, were not compared in this study, therefore no covariates required controlling.
Data exclusions	Microscopy samples with fewer than 100 parasites were excluded.<br>One day 60 scRNA-seq sample was excluded due to the poor quality of RNA capture per cell.<br>Cell transcriptomes were excluded from the analysis of remaining samples if they were determined to contain transcripts of multiple cells or from inviable cells.
Replication	Two cattle were infected for biological replicates.
Randomization	Two cattle were infected with the same parasite sample with no comparisons and so randomization was not required.
Blinding	Blinding was used with performing microscopy analysis of the flagella length and KN counting only. Blinding was not used when performing scRNA-seq analysis.

Reporting for specific materials, systems and methods

We require information from authors about some types of materials, experimental systems and methods used in many studies. Here, indicate whether each material, system or method listed is relevant to your study. If you are not sure if a list item applies to your research, read the appropriate section before selecting a response.

Materials & experimental systems

n/a	Involved in the study
☐	☒ Antibodies
☐	☒ Eukaryotic cell lines
☒	☐ Palaeontology and archaeology
☐	☒ Animals and other organisms
☒	☐ Clinical data
☒	☐ Dual use research of concern
☒	☐ Plants

Methods

n/a	Involved in the study
☒	☐ ChIP-seq
☒	☐ Flow cytometry
☒	☐ MRI-based neuroimaging

Antibodies

Antibodies used

Anti-PAD1

Validation

anti-PAD1 : For detection of Trypanosoma brucei PAD1 protein by immunofluorescence, mouse, previously validated Dean, S., Marchetti, R., Kirk, K. & Matthews, K. R. A surface transporter family conveys the trypanosome differentiation signal. Nature 459, 213–217 (2009). antiPAD1 antibody was used at 1/1000 dilution. The antibody is not commercially available and was purified for the lab by Eurogentec from immunised rabbit serum.

Secondary antibody use was Invitrogen Alexa Fluor 488 goat anti-rabbit IgG A11088 lot 2147635. Used at 1:1,000 dilution.

Eukaryotic cell lines

Policy information about cell lines and Sex and Gender in Research

Cell line source(s)

Cell lines were Trypanosoma brucei brucei Antat 1.1 90:13, provided by the Matthews Lab group, University of Edinburgh.

Authentication

Cell line was cultured in the antibiotics each is stated to be resistant to in order to authenticate prior to beginning experiments.

Mycoplasma contamination

No mycoplasma testing was performed

Commonly misidentified lines
(See ICLAC register)

N/A

Animals and other research organisms

Policy information about studies involving animals; ARRIVE guidelines recommended for reporting animal research, and Sex and Gender in Research

Laboratory animals

Two male post-weaning Holstein Friesian calves aged 4-6 months

Wild animals

N/A

Reporting on sex

Sex was not considered in this study.

Field-collected samples

N/A

Ethics oversight

Animal experiments were carried out in the Large Animal Research and Imaging Facility at the Roslin Institute, University of Edinburgh, under United Kingdom Home Office Project License number PE854F3FC. Studies were approved by the Roslin Institute (University of Edinburgh) Animal Welfare and Ethical Review Board (study numbers L447 and L475). Care and maintenance of animals complied with University regulations and the Animals (Scientific Procedures) Act (1986; revised 2013).

Note that full information on the approval of the study protocol must also be provided in the manuscript.

Plants

Seed stocks	N/A
Novel plant genotypes	N/A
Authentication	N/A
